# Generating Survival Times Using Cox Proportional Hazards Models with Cyclic and Piecewise Time-Varying Covariates

**DOI:** 10.1007/s12561-020-09266-3

**Published:** 2020-01-25

**Authors:** Yunda Huang, Yuanyuan Zhang, Zong Zhang, Peter B. Gilbert

**Affiliations:** 1 Vaccine and Infectious Disease Division, Fred Hutchinson Cancer Research Center, 1100 Fairview Ave. North, Seattle, WA 98109 USA; 2 Department of Global Health, University of Washington, Seattle, WA 98105 USA; 3 Department of Computer Science, Carnegie Mellon University, 5000 Forbes Avenue, Pittsburgh, PA 15213 USA; 4 Department of Biostatistics, University of Washington, Seattle, WA 98195 USA

**Keywords:** Correlates of risk, Joint modeling of longitudinal and survival data, Survival data simulations, Time-dependent covariate, Zero-protection threshold

## Abstract

Time-to-event outcomes with cyclic time-varying covariates are frequently encountered in biomedical studies that involve multiple or repeated administrations of an intervention. In this paper, we propose approaches to generating event times for Cox proportional hazards models with both time-invariant covariates and a continuous cyclic and piecewise time-varying covariate. Values of the latter covariate change over time through cycles of interventions and its relationship with hazard differs before and after a threshold within each cycle. The simulations of data are based on inverting the cumulative hazard function and a log link function for relating the hazard function to the covariates. We consider closed-form derivations with the baseline hazard following the exponential, Weibull, or Gompertz distribution. We propose two simulation approaches: one based on simulating survival data under a single-dose regimen first before data are aggregated over multiple-dosing cycles and another based on simulating survival data directly under a multiple-dose regimen. We consider both fixed intervals and varying intervals of the drug administration schedule. The method’s validity is assessed in simulation experiments. The results indicate that the proposed procedures perform well in generating data that conform to their cyclic nature and assumptions of the Cox proportional hazards model.

**Electronic supplementary material:**

The online version of this article (10.1007/s12561-020-09266-3) contains supplementary material, which is available to authorized users.

## Introduction

**Fig. 1:**
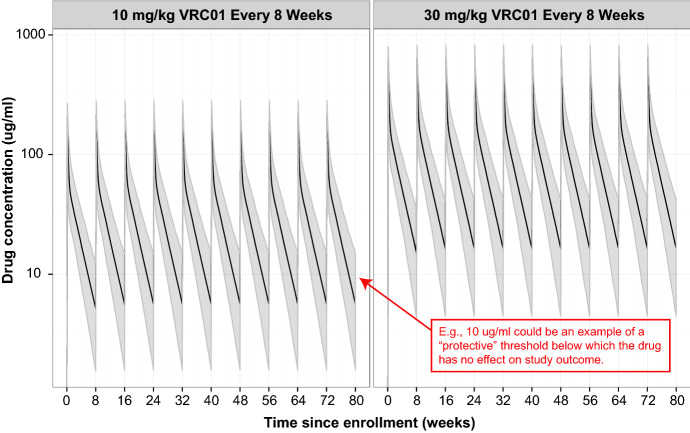
Illustration—simulated VRC01 serum concentration over time following ten 8-weekly IV infusions at 10 mg/Kg and 30 mg/Kg dose levels with perfect study adherence, according to the pharmacokinetics model described in Huang et al. [2]. Solid lines are the medians; shaded areas are the 2.5% and 97.5% percentiles of the concentrations over 1000 simulated datasets. A body weight of 74.5 Kg is used in the simulations

Time-to-event outcomes with cyclic time-varying covariates are frequently encountered in biomedical studies that involve multiple or repeated administrations of an intervention. For example, the plasma concentration of a drug taken orally daily to prevent a certain infection would usually fluctuate on a daily cycle, and it is often of interest to identify whether and how the cyclic drug concentration associates with the hazard of infection. In the two harmonized Antibody Mediated Prevention (AMP) Phase 2b efficacy trials (ClinicalTrials.gov #NCT02716675 & #NCT02568215), more than 4500 HIV-uninfected participants at high risk for acquiring HIV infection are randomized to receive 10 infusions every 8 weeks of either VRC01 or placebo and followed for 80 weeks for the study endpoint of HIV infection [1]. VRC01 is a monoclonal antibody that has been shown to neutralize most strains of the HIV virus in laboratory studies, and the AMP trials will test whether VRC01 reduces the rate of HIV infection compared to placebo. The concentration of VRC01 in participants’ blood samples, which we refer to as “drug concentration,” changes continuously and cyclically over time. As illustrated in Fig. 1, the drug concentration typically peaks within hours after an infusion, followed by a decline over weeks of time and may reach below a clinically protective threshold, *s* [2–4].

In the context of drug concentration being a potential biomarker that predicts the risk of infection, *s* is referred to as the zero-protection threshold. This implies that, during periods when drug concentration is below *s*, the individual receives no protection from the drug. A simulation model was previously developed to address the same issue, for a different context of studying time-independent biomarkers as correlates of cumulative outcome risk [5]. The primary objective of the AMP trials is to evaluate the efficacy of VRC01 (vs. placebo) to prevent HIV infection at dose levels of 10 mg/Kg and 30 mg/Kg. A key secondary objective assesses the association of the current value of VRC01 serum concentration (or other anti-viral functional biomarker) with the instantaneous rate of HIV infection. Such time-dependent survival analysis is desirable to aid HIV vaccine development by setting a benchmark biomarker value for the required potency of a vaccine-induced immune response to putatively achieve a high level of protection against HIV infection. Findings from such analysis will thus help define study endpoints in phase 1 and 2 trials that vet candidate HIV vaccines for their potential efficacy [1].

For joint modeling of longitudinal biomarker data and time-to-event data (e.g., [6–8]), simulation studies are often needed in the method development for the analyses of such data. An essential starting point is to produce simulated survival times from a known data-generating process [9–13]. For continuous covariates, to our knowledge previous work has been limited to simulating event times that the time-varying covariates follow a simple linear relationship with time and/or log-transformed time [14–16], or the covariates change at integer-valued steps of the time scale [17] throughout the entire follow-up period. Such data-generating processes are only appropriate when individuals are uniformly exposed to risk of acquiring the survival outcome at each unit of time (e.g., oral daily dose of the same drug amount). Therefore, new or extensions of these methods are needed for settings like the AMP trials with a cyclic and piecewise time-varying covariate.

Cox proportional hazards (PH) regression models are the most common approach for evaluating the association of covariates, including time-varying covariates with survival outcomes. The objective of this paper is to develop methods for the generation of survival times that follow a Cox PH model with time-invariant covariates, as well as a cyclic and piecewise time-varying covariate. We generate time-to-event data based on inverting the cumulative hazard function and a log link function for relating the hazard function to the time-varying and time-invariant covariates. We provide closed-form derivations for simulating time-to-event data with the baseline hazard following three commonly used distributions: exponential, Weibull, and Gompertz, all of which satisfy the PH assumptions with the Cox regression model [18] . We propose two simulation approaches. The first approach is based on simulating survival data under a single-dose regimen before such data are aggregated over multiple-dose intervals; the second approach is based on simulating survival data directly under a multiple-dose regimen. Under the latter approach, we also provide derivations for simulating time-to-event data from studies with varying drug administration intervals to accommodate variable visit windows and possible missed visits.

The paper is structured as follows. In Sect. 2, we introduce notations and assumptions, followed by descriptions of the single-dose and multiple-dose approaches for simulating survival times. For the single-dose approach, we provide, under the zero-protection model, details of the closed-form derivations of the baseline hazard following an exponential distribution in the main text. Derivations for Weibull- and Gompertz-distributed baseline hazard are presented in the Appendices. For the multiple-dose approach, we provide details of the derivations assuming a monotonic relationship between the time-varying covariate and the survival outcome within each dosing cycle in the main text. Extensions incorporating the zero-protection model and varying drug administration intervals are provided in the Online Appendices. In Sect. 3, we describe three simulation experiments to assess the developed methods with application to the AMP CoR study. Conclusions are provided in Sect. 4.

## Methods

For concreteness, we describe notations and methods in the context of the AMP trials. However, the same data-generating concepts can be generalized to other applicable biomedical settings with repeated drug administrations.

### Notation

Let event time, *t*, be time (in days) from study enrollment (i.e., first study administration) to HIV-1 infection, and $\tau$ the final study follow-up visit time. Suppose a maximal number of *M* infusions are planned in the study ($M =10$ for AMP) and *m* be the number of infusions one actually received, where $m\le M$ due to possible missed infusions or early dropout. Let $D_1 \dots D_m$ be the actual dose infusion visit times since enrollment with $0 = D_1< \dots < D_m \le \tau$. Let $I_1$, $I_2$, $\dots$, $I_{m-1}$ be the $m-1$ infusion interval lengths (in days) between the *m* infusions, and $I_m$ the interval between the last infusion and the end of follow-up in that $I_k=D_{k+1} -D_k$, $k=1$, 2, ..., *m*-1, and $I_m=\tau -D_m$.

The hazard of HIV-1 infection, *h*(*t*), is modeled as a function of time-invariant covariates and a time-varying covariate according to the Cox PH model as

1 $$\begin{aligned} h(t|x,z(t)) = h_{0}(t)\exp (\beta z(t)+\eta ^\prime x), \end{aligned}$$

where *z*(*t*) denotes the time-varying covariate, whose value changes over the duration of the follow-up time, while its association with the hazard of the outcome stays constant as denoted by the regression coefficient $\beta$; *x* denotes the time-invariant covariates, and $\eta$ is the vector of regression coefficients associated with the vector of fixed covariates *x*. $h_{0}(t)$ is the baseline hazard function, i.e., the hazard function of the outcome for those subjects with $x=0$ and $z(t)=0$. In addition, let $t_s$ be the time (in days) since the most recent infusion when drug concentration reaches the zero-protection threshold, *s*.

### Cyclic and Piecewise Time-Varying Covariate

Under the zero-protection threshold model, we define the time-varying covariate as time since the most recent infusion or $t_s$ in a cyclic and piecewise manner:

2 $$\begin{aligned} z(t) = {\left\{ \begin{array}{ll} t-D_k &{} \text {if } D_k< t\le D_{k+1}\, \& \, t-D_k \le t_s, \text { } k=1, 2, \ldots , m-1,\\ t-D_m &{} \text {if } D_m < t\le \tau \, \& \, t-D_m \le t_s,\\ ts &{} \text {otherwise}.\\ \end{array}\right. } \end{aligned}$$

Although *z*(*t*) may be defined directly as the drug concentrations over time, one advantage of the above definition is the easy interpretation of $\beta$ in Eq. (1) as the per-day change effect on log-hazard before $t_s$ is reached within each drug administration cycle. Intuitively, $\beta$ is $\ge 0$ if the risk of infection with respect to *z*(*t*) is expected to be nondecreasing over time within each cycle. In other words, we consider *z*(*t*) as a proxy of the drug concentration at time *t* because after each infusion, drug concentrations are expected to change with time in a monotonic fashion. For example, for drug concentrations that follow a log-linear relationship with time, as specified by a one-compartment pharmacokinetics (PK) model with a single decay rate, or for drug concentrations that follow a bi-exponential two-compartment PK model with a brief distribution phase but a much longer elimination phase (as shown in Fig. 1), the effect of drug concentration on log-hazard is measured by simply rescaling $\beta$ by the elimination decay rate. This relationship is expected to be held for many monoclonal antibodies that exhibit the described pharmacokinetic patterns (see review in, e.g., [19]).

Another advantage of this definition of *z*(*t*) is the generalizability of the derivations described in the following sections without being constrained to a specific nonlinear PK model of drug concentration over time, while also sidestepping the issue of not having a closed-form derivation of the survival time for more complex nonlinear PK models. The reason why *z*(*t*) takes the value of $t_s$ after drug concentration reaches below *s* is to ensure that, beyond $t_s$ within each drug administration cycle, the hazard of individuals who received the drug does not keep changing at the rate of $\exp (\beta )$ but maintains at the same level as that of individuals who did not receive the drug. This tactic avoids the need to impose a different value of $\beta$ when the effect of the time-varying covariate changes after $t_s$ under the zero-protection threshold model.

In reality, $t_s$ could differ across individuals. For simplicity and faster computation, an average $t_s$ can be used in the actual simulation of survival times. For example, based on the population PK model of VRC01 described in Huang et al. [2] , we estimate that $t_s=57$ days for the 10 mg/Kg dose group, and $t_s=81$ days for the 30 mg/Kg dose group with $s=5.0$ mcg/mL, a level of VRC01 concentration that is hypothesized to confer protection against HIV infection [20–23]. This implies that the instantaneous hazard remains constant after 57 and 81 days, respectively, in the low- and high-dose groups of the AMP trials. This ensures meaningful simulated survival time to account for the wide infusion visit window in AMP (− 1 week to + 7 weeks around the target 8-weekly infusion visits) and for individuals whose infusion intervals are great than 8 weeks due to missed infusions.

### Assumptions

The following assumptions are used in the derivations for the single- and multiple-dose approaches described below.

- The effect of both the time-invariant and time-varying covariates on hazard is multiplicative (i.e., the PH assumption).
- $\beta$ is a time-invariant coefficient in Eq. (1). This implies that the association between *z*(*t*) and hazard does not change between cycles (i.e., the cycle-invariant assumption).
- Under the zero-protection threshold model, the instantaneous hazard at $t_s$ within each cycle is assumed to be $h(t=D_k+t_s|x,z(t))=h_0(t_s)\exp (\beta t_s+\eta ^\prime x) = \lambda _p(x)$, $k=1$, 2, ..., *m*, where $\lambda _p(x)$ indicates the hazard rate in the control group where no association of the drug with survival is expected to be observed. Of note, $\lambda _p(x)$ is allowed to vary with *x* if incorporating between-individual variability due to time-invariant covariates is desirable in the simulated datasets.

### Simulating Survival Times

As shown in Eq. (1), the Cox model is formulated through the hazard function. Therefore, the simulation of appropriate survival times for this model needs further manipulation based on the relationship between the hazard function and the covariate as discussed in [9–12, 14–17]. The translation of the regression coefficients from hazard to survival time is relatively easy if the baseline hazard function is constant with $h_{0}(t)=\lambda$, $\lambda >0$. In this case, the cumulative hazard function of model (1) is given by:

3 $$\begin{aligned} H(t|x,z(t))= \int ^{t}_{0}{\lambda \exp (\beta z(u)+\eta ^\prime x)\,\mathrm{{du}}}. \end{aligned}$$

Because of the survival function of the above model, $S(t|x,z(t))=\exp (-H(t|x,z(t)))$ follows the standard uniform distribution U(0,1), [9, 11, 12] have demonstrated that a survival time, *T*, can be generated by inverting the cumulative hazard function via $T=H^{-1}(-\log (U))$, where $U \sim U(0,1)$.

In the following, we extend the work of Austin [14] to accommodate both time-invariant covariates, *x*, and a continuous time-varying covariate, *z*(*t*). Importantly, the values of *z*(*t*) change over time in a cyclic form and the association between *z*(*t*) and survival changes in a piecewise manner within each cycle.

#### Single-Dose Approach

The single-dose approach considers simulating survival data over one-dose interval as a first step before such data are aggregated over multiple-dose intervals. Instead of having the same continuous relationship with *t* throughout the entire follow-up time as described in Austin [15], *z*(*t*) in our case changes at $t_s$ within each drug administration cycle, as shown in Eq. (2). In the following, we describe the steps to simulate survival times after a single dose, by inverting the cumulative hazard function. We show derivations in details for Cox models with an exponential baseline hazard; details for the Weibull and Gompertz distributions are reported in Online Appendices A1 and A2, respectively.

For exponentially distributed baseline hazard, $h_{0}(t)=\lambda$, *t* actually follows the Gompertz distribution with a scale parameter of $\lambda \exp (\eta ^\prime x)$ and a shape parameter of $\beta$. Therefore, if $t \le t_s$, the event time can be generated as

4 $$\begin{aligned} T = \frac{1}{\beta } \log \left( 1+\frac{\beta (- \log (u))}{\lambda \exp (\eta ^\prime x)} \right) \text{, } \text{ if } -\log (u) & \frac{\lambda \exp (\eta ^\prime x)}{\beta }\left[ \exp (\beta t_s)-1\right] , \end{aligned}$$

where *u* is the realization of a *U*(0, 1) random variable. The detailed derivations are provided in Online Appendix A0 and follow similar steps as described in Austin [15] for Gompertz-distributed event times.

If $t > t_s$, the cumulative hazard function is equal to

$$\begin{aligned} H(t,x,z(t))= & {} \int ^{t_s}_{0}{\lambda (\beta u + \eta ^\prime x)\,\mathrm{{du}}}+ \int ^{t}_{t_s}{ \lambda \text{ exp }(\beta t_s + \eta ^\prime x),\mathrm{{du}}} \\= & {} \lambda \text{ exp }(\eta ^\prime x)\left( \frac{1}{\beta }(\text{ exp }(\beta t_s)-1) + (t-t_s) \text{ exp }(\beta t_s)\right) . \end{aligned}$$

Consequently, the inverse cumulative hazard function is

$$\begin{aligned} H^{-1}(v) = \frac{v}{\lambda \text{ exp }(\beta t_s +\eta ^\prime x)} + \frac{1- \text{ exp }(\beta t_s)}{\beta \text{ exp }(\beta t_s)} + t_s. \end{aligned}$$

Therefore, an event time can be generated as

5 $$\begin{aligned} T = \frac{-\text{ log }(u)}{\lambda \text{ exp }(\beta t_s +\eta ^\prime x)} + \frac{1- \text{ exp }(\beta t_s)}{\beta \text{ exp }(\beta t_s)} + t_s, \text{ if } -\text{ log }(u) \ge \frac{\lambda \text{ exp }(\eta ^\prime x)}{\beta }\left[ \text{ exp }(\beta t_s)-1\right] , \end{aligned}$$

where *u* is the realization of a *U*(0, 1) random variable.

In summary, in order to simulate survival times under a zero-protection threshold model after a single dose is given, a random uniform sample, *u* , is first simulated and the survival time takes the form in Eq. (4) if $-\text{ log }(u) < \frac{\lambda \text{ exp }(\eta ^\prime x)}{\beta }\left[ \text{ exp }(\beta t_s)-1\right]$, or the form in Eq. (5), otherwise.

After the single-dose survival time according to the exponential, Weibull-, or Gompertz-distributed baseline hazard is simulated as described above or in the Online Appendix, the survival time after multiple doses can be simulated as follows:

1. Simulate the actual infusion visit times (since enrollment), $D_1< \dots < D_m$ for each individual’s *m* infusions  (e.g., [24]). Consequently, the infusion intervals can be calculated as $I_k=D_{k+1} -D_k$, $k=1$, 2, ..., $m-1$, and $I_m=\tau - D_m$. Infusion visit windows and possible missed infusions and/or permanent infusion discontinuations could be considered here. For example, the probability of a missing visit can be specified for each infusion for different scenarios of adherence level. In the case of the AMP study, the target visit date of each subsequent infusion is relative to the immediately previous infusion visit. Therefore, for AMP, the actual infusion visit times will need to be simulated sequentially and an uniform distribution could be used to simulate the visit time of an infusion to be between a window of, say 51 days and 105 days after the previous infusion visit date;
2. For each individual, independently simulate the single-dose survival time $T_1$, $T_2$, ..., $T_m$ for each of the *m* infusion intervals according to Eqs. (4) and (5);
3. If all $T_k> I_k$, $k=1$, 2, ..., *m*, then the final multiple-dose survival time of this uninfected individual is censored at $S=\sum \nolimits _{i=1}^{m}I_i$. Otherwise, randomly pick a *k* that satisfies $T_k < I_k$, and the final multiple-dose survival time for this infected individual is $S = \sum \nolimits _{i=1}^{k-1}I_i + T_k$.

This approach guarantees that, as desired, the event time follows the same survival function within each infusion interval. In addition, the probability of infection during a given interval is not affected by the probability of the same individual not being infected in the prior infusion interval because P(infected in $I_2$) = P(infected in $I_2$ | not infected in $I_1$) = P ($T_2< D_2$ | $T_1> D_1$) = P ($T_2 < D_2$) (given that $T_k$’s are i.i.d).

#### Multiple-Dose Approach

For simulating survival time with a cyclic time-varying covariate, instead of the approach described above via aggregating survival times generated in single-dose intervals, the multiple-dose approach considers simulating survival data over multiple-dose intervals directly. The following steps can be used to generate survival times for participants receiving up to *m* doses. These steps apply when all dosing intervals, $I_1, \ldots , I_m$, are smaller than $t_s$, i.e., the next dose is always given or the study is ended before the drug concentration reaches below *s*. If some of $I_1, \ldots , I_m$ may be greater than $t_s$, then strategies that combine the cumulative hazards before $t_s$ and after $t_s$ can be employed (Online Appendix A3). Similarly, survival times are simulated by inverting the cumulative hazard function. In the following derivations, the baseline hazard is assumed to be exponentially distributed.

If $D_1 \le t<D_2$, following similar derivations shown in Online Appendix A0, *t* can be generated as

6 $$\begin{aligned} T = \frac{1}{\beta } \text{ log }\left( 1+\frac{\beta (- \text{ log }(u))}{\lambda \text{ exp }(\eta ^\prime x)} \right) \text{, } \text{ if } -\text{ log }(u) < b_1 \end{aligned}$$

where $$b_1 = \frac{\lambda }{\beta } \text{ exp }(\eta ^\prime x)\left[ \text{ exp }(\beta \times D_2)-1\right] ,$$ and $$u\sim U(0,1)$$.

If $D_2\le t < D_3$, the cumulative hazard function is equal to

$$\begin{aligned} H(t,x,z(t))= & {} \int ^{t}_{0}{\lambda \text{ exp }(\beta z(u)+\eta ^\prime x)\,\mathrm{{du}}} \\= & {} \lambda \text{ exp }(\eta ^\prime x)\int ^{t}_{0}{\text{ exp }(\beta u)\,\mathrm{{du}}} \\= & {} \lambda \text{ exp }(\eta ^\prime x)\left( \int ^{D_2}_{0}{\text{ exp }(\beta u)\,\mathrm{{du}}}+ \int ^{t}_{D_2} { \text{ exp }(\beta (u-t_2))\,\mathrm{{du}}}\right) \\= & {} \lambda \text{ exp }(\eta ^\prime x)\left( \frac{1}{\beta }(\text{ exp }(\beta \times D_2)-1) + \frac{1}{\beta }(\text{ exp }(\beta t-\beta \times D_2)-1)\right) \\= & {} \frac{\lambda }{\beta }\text{ exp }(\eta ^\prime x)\left( \text{ exp }(\beta \times D_2) + \text{ exp }(\beta t-\beta \times D_2)-2\right) . \end{aligned}$$

Consequently, the inverse cumulative hazard function is

$$\begin{aligned} H^{-1}(u) = \frac{1}{\beta } \text{ log }\left( \text{ exp }(\beta \times D_2)\left( \frac{\beta u}{\lambda \text{ exp }(\eta ^\prime x)}-\text{ exp }(\beta \times D_2)+2\right) \right) . \end{aligned}$$

Therefore, an event time can be generated as

$$\begin{aligned} T = \frac{1}{\beta } \log \left( \exp (\beta \times D_2)\left( \frac{\beta (-\log (u))}{\lambda \exp (\eta ^\prime x)}-\exp (\beta \times D_2)+2\right) \right) \text{, } \\\text{ if } a_2 \le -\log (u) < b_2, \end{aligned}$$

where

$$\begin{aligned} a_2= & {} \frac{\lambda }{\beta }\exp (\eta ^\prime x)\left( \exp (\beta \times D_2)-1\right) ,\\ b_2= & {} \frac{\lambda }{\beta }\exp (\eta ^\prime x)\left( \exp (\beta \times I_1)+ \exp (\beta \times I_2)-2\right) ,\text { and} \end{aligned}$$

*u* is the realization of a *U*(0,1) random variable.

Similarly, for $D_{k}\le t < D_{k+1}$, $k=2, \ldots , m-1$, the cumulative hazard function is

$$\begin{aligned} H(t,x,z(t)) = \frac{\lambda }{\beta }\exp (\eta ^\prime x)\left[ \sum \limits _{i=2}^{k}\exp (\beta \times I_{i-1}) + \exp (\beta t-\beta \times D_{k})-k\right] , \end{aligned}$$

and the inverse cumulative hazard function is

$$\begin{aligned} H^{-1}(u) = \frac{1}{\beta } \text{ log }\left( \exp (\beta \times D_{k})\left( \frac{\beta u}{\lambda \exp (\eta ^\prime x)}-\sum \limits _{i=2}^{k}\exp (\beta \times I_{i-1})+k\right) \right) . \end{aligned}$$

Therefore, an event time can be generated as

7 $$\begin{aligned} T=\frac{1}{\beta } \text{ log }\left( \exp (\beta \times D_{k})\left( \frac{\beta (-\log (u))}{\lambda \exp (\eta ^\prime x)}-\sum \limits _{i=2}^{k}\exp (\beta \times I_{i-1})+k\right) \right) \text{, } \\\text{ if } a_k \le -\log (u) < b_k, \end{aligned}$$

where

$$\begin{aligned} a_k= & {} \frac{\lambda }{\beta }\exp (\eta ^\prime x)\left( \sum \limits _{i=2}^{k}\exp (\beta \times I_{i-1})-(k-1)\right) ,\\ b_k= & {} \frac{\lambda }{\beta }\exp (\eta ^\prime x)\left( \sum \limits _{i=2}^{k+1}\exp (\beta \times I_{i-1})-k\right) , \text { and} \end{aligned}$$

*u* is the realization of a *U*(0, 1) random variable.

Lastly, if $t\ge D_m$, an event time can be generated as

8 $$\begin{aligned} T=\frac{1}{\beta }\log \left( \exp (\beta \times D_{m})\left( \frac{\beta (-\log (u))}{\lambda \text{ exp }(\eta ^\prime x)}-\sum \limits _{i=2}^{m}\exp (\beta \times I_{i-1})+m\right) \right) \text{, } \\\text{ if } -\log (u) \ge b_m, \end{aligned}$$

where $b_m = \frac{\lambda }{\beta }\text{ exp }(\eta ^\prime x)\left( \sum \nolimits _{i=2}^{m}\exp (\beta \times I_{i-1})-(m-1)\right)$ and *u* is the realization of a *U*(0, 1) random variable.

In summary, the infusion times, $D_1<\dots <D_m$, for each participant and a random uniform variate *U* are first simulated. Then, for each $k=1, \ldots , m-1$, $a_k$ and $b_k$ are calculated, where $a_1=0$ and $a_{k+1}=b_k$. The survival time takes the form in Eq. (6) if $-\log (u) < b_1$, or the form in Eq. (7) if $a_k \le -\log (u) < b_k$, or the form in Eq. (8) if $-\log (u) \ge b_9$.

## Applications

We next apply the described survival data-generating approaches and evaluate their validity in simulation experiments. These simulations are motivated by the AMP trials in the context of HIV infection; however, the described procedures can be generalized to other biomedical applications. In the context of HIV infection in healthy adults, previous HIV vaccine efficacy trials have found support for the assumption of a constant hazard over time in the placebo group [25–30]. Therefore, we chose the exponential baseline hazard function in the following illustrations.

In the first experiment, the single-dose approach is used to simulate survival data for 1000 AMP-like trials, each with $n=1500$ participants in each of the 10 mg/Kg VRC01, 30 mg/Kg VRC01 and placebo groups. Within each trial, the time-varying covariate (i.e., time since infusion) is associated with the survival outcome (i.e., time to HIV infection in days) according to Eq. (1) with $\beta =0.03$ and $\eta =0$ for both dose groups. In addition, *z*(*t*) takes the piecewise form as described in Eq. (2) with a zero-protection threshold $s=5$ mcg/mL. Consequently, the baseline hazard $h_{0}(t)=$ daily HIV incidence rate divided by $\text {exp}(\beta t_s)$, where $t_s=57$ and $t_s=81$ for the low- and high-dose groups, respectively, to ensure the same baseline HIV infection rate beyond $t_s$ in the two dose groups. These parameter values indicate that, before an individual’s drug concentration reaches 5 mcg/mL, the hazard ratio over a 28-day period is exp$(28\times 0.03)=2.31$, but the rate of infection remains constant ($=0.04$/year) once the individual’s drug concentration falls below 5 mcg/mL. We consider two study adherence levels: the high and medium adherence scenarios assume the probability of missing a given infusion is 2% and 10% for each of the 10 infusions, and are simulated based on random draws from binomial distributions with success probabilities 0.02 and 0.1, respectively. More details in the simulations of such AMP-like study setup can be found in Zhang et al. [8].

**Fig. 2:**
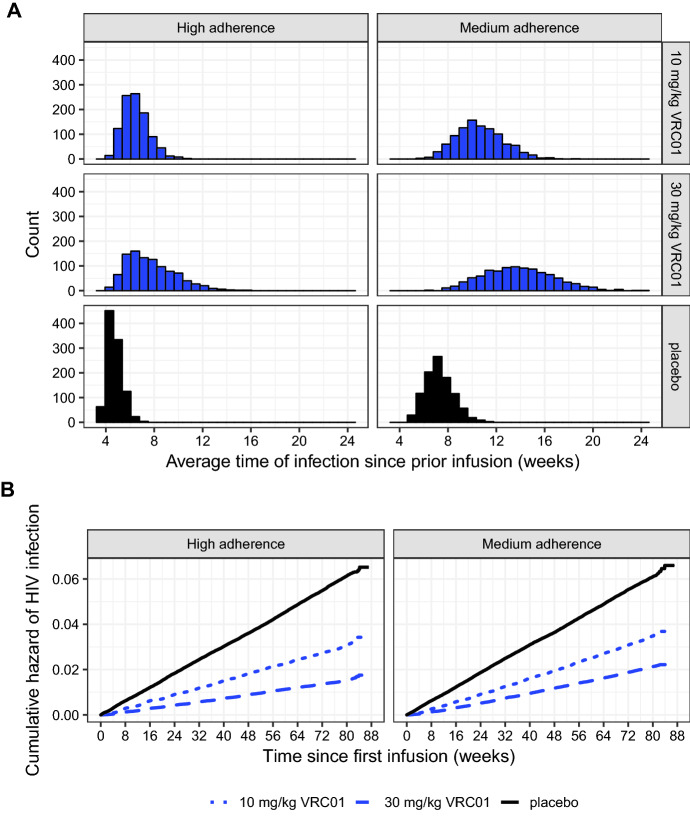
Distributions of simulated event times since prior infusion (**a**) and cumulative hazard of HIV infection since the first infusion (**b**) under imperfect study adherences in AMP-like trials. The single-dose approach is used in these simulations of 1000 trials, each with a total of $n=4500$ participants randomized to receive ten 8-weekly infusions of 10 mg/Kg VRC01, 30 mg/Kg VRC01, or placebo in a 1:1:1 ratio. The high and medium adherence scenarios assume 2% and 10% of infusion visits missed, respectively. Additional assumptions are as follows: annual HIV incidence rate $=4\%$ in the placebo group, $\beta =0.03$ or HR$=2.32$ per 28 days for both VRC01 dose groups, and zero-protection concentration threshold $s=$ 5 mcg/mL

We expect three patterns in the simulated data. First, the low-dose group should have higher risk of infection than the high-dose group. This is because drug concentrations in the former group on average are expected to reach the zero-protection threshold, 5 mcg/mL in a shorter time or, in other words, the lower-dose group is expected to have a smaller $t_{s=5 \,\mathrm{{mcg/mL}}}$ than the higher-dose group, although the two dose groups do have the same risk (due to having the same $\beta =0.03$) until their respective $t_{s=5\, \mathrm{{mcg/mL}}}$ time points within each dosing cycle. Second, a lower risk of infection should be associated with a better study adherence due to less missed infusions and less follow-up time with concentration below the zero-protection threshold $s=5$ mcg/mL. Third, a shorter duration between time of infection and prior infusion should occur with better study adherence due to shorter average infusion intervals when there are less missed infusions, although a smaller number of infections do occur with a better study adherence. As shown in Fig. 2, all these patterns are confirmed. In addition, the same patterns are observed when the sample size is reduced to $n=700$/group (Online Appendix A4).

**Fig. 3:**
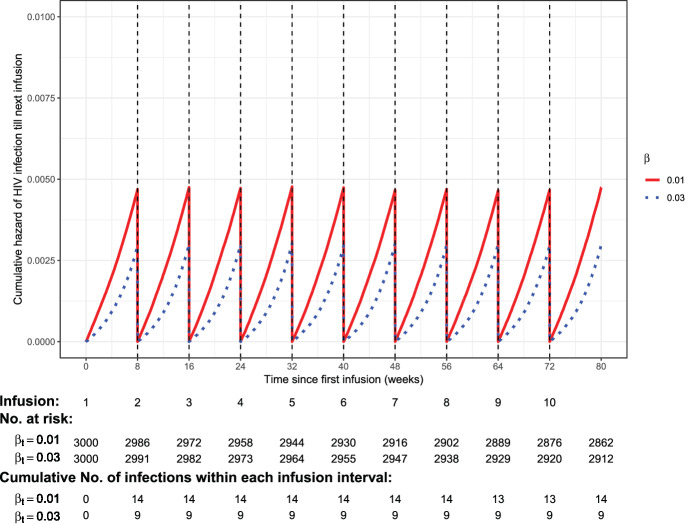
Cumulative hazard of HIV infection within each infusion interval following ten 8-weekly IV infusions of VRC01 under perfect study adherence in a simulated trial of 3000 VRC01 recipients. Red lines are for $\beta =0.01$ or $HR=1.32$ per 28 days; blue lines are for $\beta =0.03$ or HR$=2.32$ per 28 days (Color figure online)

In the second experiment, the multiple-dose approach is used to simulate AMP-like trials under perfect study adherence scenarios with $\eta =0$ and $h_{0}(t)=$ daily HIV incidence rate divided by $\text {exp}(\beta \times 56)$. Each trial includes $n=1500$ VRC01 recipients in each of the 10 mg/Kg and 30 mg/Kg dose groups. Two $\beta$ values, 0.01 and 0.03, are considered in order to verify how risk of infection varies by $\beta$ within each dose group. Similar to the first experiment, the two dose groups share the same *beta* value under each scenario. Figure 3 shows that the probability of HIV infection within each 8-weekly infusion cycle is smaller as $\beta$ gets larger. This pattern is also expected because a higher $\beta$ indicates a larger association of the biomarker with reduced risk of infection. In addition, as desired, the rate of HIV infection increases over time (as concentration gets lower) within each infusion cycle, and the pattern remains the same over all cycles under the ‘cycle-invariant’ assumption described in Sect. 2.3. The same patterns are observed when the sample size is reduced to $n=700$/group (Online Appendix A4).

In the third experiment, we expand the first experiment with a total of four $\beta$ values in both the high and medium adherence scenarios. We evaluate the empirical characteristics of ${\hat{\beta }}$. Specifically, within each simulated trial of $n=3000$ VRC01 recipients, besides the cyclic and piecewise time-varying covariate, *z*(*t*) defined in (Sect. 2.2), we construct an indicator function, $I(z(t)\le t_s)$, as another time-dependent covariate. We then use a Cox proportional hazards regression model to regress time-to-infection on the product of *z*(*t*) and $I(z(t)\le t_s)$; $\beta$ is the coefficient of this interaction term with its interpretation preserved as the per-day change in log-hazard prior to $t_s$ within each dosing interval. As illustrated in Table 1, the maximum partial likelihood estimator of $\beta$ is close to zero empirical bias and Wald-type 95% confidence intervals for $\beta$ with the robust Huber sandwich variance estimator achieves nominal coverage as desired. This further confirms that data simulated using our proposed method maintain the intended nature and effect size of the cyclic and piecewise time-varying covariate.

**Table 1:** Empirical characteristics of ${\hat{\beta }}$

Adherence	True $\beta$	Mean of ${\hat{\beta }}$	RBias%	RRMSE%	Coverage%
High	0.01	0.01	0	1.0	95.0
	0.02	0.0197	$-$ 1.5	0.5	95.4
	0.03	0.0286	$-$ 4.7	0.3	95.2
	0.04	0.0384	$-$ 4.0	0.5	95.7
Medium	0.01	0.0097	$-$3.0	1.0	95.8
	0.02	0.0192	$-$ 4.0	0.5	96.0
	0.03	0.0291	$-$ 3.0	0.3	95.1
	0.04	0.0393	$-$ 1.8	0.5	95.2

Under each scenario, the single-dose approach is used to simulate 1000 trials each with a total of $n=3000$ participants receiving 10 mg/Kg VRC01 or 30 mg/Kg VRC01. $\beta$ indicates the per-day increase in log-hazard of HIV infection for both dose groups. ${\hat{\beta }}$ is estimated using a Cox proportional hazards model as described in Sect. 3. Reported are mean of ${\hat{\beta }}$, relative bias, RBias $=\frac{1}{K}\sum ^{B}_{k=1}(\frac{{{\hat{\beta }}}_k - \beta }{\beta }) \times 100$, relative root mean squared error, RRMSE $=\root \of {\frac{1}{K}\sum ^{B}_{k=1}(\frac{{{\hat{\beta }}}_k - \beta }{\beta })^{2}} \times 100$, and coverage probability, CP$=$proportion of datasets with Wald-type 95% confidence intervals including the true value of the parameter $\beta$. The high and medium adherence scenarios assume 2% and 10% of infusion visits missed, respectively. Annual zero-protection HIV incidence rate is $4\%$ with a zero-protection concentration threshold $s=$ 5 mcg/mL

In addition, our proposed methods have been applied to simulate survival data in the evaluation of pharmacokinetics marker correlates of outcome [31]. popPK models were used to estimate the marker value over time [2]. Satisfactory performance was observed in terms of type I error and statistical power to detect as statistically significant the hazard ratio of HIV infection associated with the pharmacokinetics marker.

## Conclusions

In this paper, we considered simulating event time data with a continuous time-varying and piecewise covariate. The values of the covariate vary with time through multiple repetitive cycles, and its association with survival changes differently before and after a threshold within each cycle. The latter particularly accommodates settings with a zero-protection biomarker threshold, above which the drug provides a varying level of protection depending on the biomarker level, but below which the drug provides no protection. We proposed two simulation approaches: one based on simulating survival data under a single-dose regimen first before data are aggregated over multiple doses and another based on simulating survival data directly under a multiple-dose regimen. The derivations of the former are more straightforward for handling different event time distributions and can be more easily extended to data models with multiple protection threshold values within a cycle. The derivations of the latter are more compact, and simulations are generally faster than those based on the former approach. The latter approach is also more flexible to be extended to data models with cycle-specific *z*(*t*) functions.

Motivated by the AMP data example, we considered that the time-varying covariate values (i.e., log-transformed drug concentrations) change linearly with time before the protection threshold is reached. Similar derivations can be carried out for covariates that follow a more complex nonlinear relationship with time. In those cases, approximations may be needed in the inversion of the cumulative hazard function. The validity of our proposed methods was assessed in multiple simulation experiments. The results indicate that the proposed procedures perform well in producing data that conform to their cyclic and piecewise and the effect size of the time-varying covariate under a Cox model. An extension can be considered to add the number of doses as another time-dependent covariate. Consequently, the ‘cycle-invariant’ assumption about the effect of the time-varying covariates not changing between cycles can hence be relaxed. Lastly, for drugs that do not satisfy the ‘cycle-invariant’ assumption, different $\beta$ coefficients can be assumed for each cycle and derivations of the simulation procedure based on the multiple-dose approach can be similarly extended for such data models.

## Electronic supplementary material

Below is the link to the electronic supplementary material.

Supplementary materials listing A0: single-dose approach assuming exponential distribution of baseline hazard, t ≤ ts. A1: single-dose approach assuming Weibull distribution of baseline hazard. A2: single-dose approach assuming Gompertz distribution of baseline hazard. A3: multiple-dose approach assuming imperfect infusion adherence. A4: illustration of the single-dose and multiple-dose approaches with alternative sample sizes, complementing Figures 2 and 3 in the main text. Electronic supplementary material 1 (PDF 921 kb)

## Data Availability

Software in the form of R code is available at https://github.com/lilyzhangyuanyuan/AMP-survival-simulation.
