# Supplementary material for: Generating Survival Times Using Cox Proportional Hazards Models with Cyclic and Piecewise Time-Varying Covariates
Source: Stat Biosci. 2020 Jan 25;12(3):324–39. doi: 10.1007/s12561-020-09266-3 (PMC7223425; doi:10.1007/s12561-020-09266-3)
Supplement: Supplementary file 1 — Supplementary materials listing A0: single-dose approach assuming exponential distribution of baseline hazard, t ≤ ts. A1: single-dose approach assuming Weibull distribution of baseline hazard. A2: single-dose approach assuming Gompertz distribution of baseline hazard. A3: multiple-dose approach assuming imperfect infusion adherence. A4: illustration of the single-dose and multiple-dose approaches with alternative sample sizes, complementing Figures 2 and 3 in the main text. Electronic supplementary material 1 (PDF 921 kb) [file 12561_2020_9266_MOESM1_ESM.pdf]

Statistics in Biosciences

Generating survival times using Cox proportional hazards models with cyclic and piecewise time-varying covariates – Supplementary Materials

Yunda Huang[a,b,*], Yuanyuan Zhang[a], Zong Zhang[c], Peter B. Gilbert[a,d]

[a] *Vaccine and Infectious Disease Division, Fred Hutchinson Cancer Research Center,*

[b] *Department of Global Health, University of Washington,* [c] *Interlake High School,* [d] *Department of Biostatistics, University of Washington, Washington, USA*

yunda@fredhutch.org

Appendix A0: single-dose approach assuming Exponential distribution of baseline hazard, $t \leqslant t_s$

Survival times after a single dose are simulated by inverting the cumulative hazard function for Cox models with an Exponential baseline hazard, $h_0(t) = \lambda$, following similar steps described in Austin (2012).

*To whom correspondence should be addressed.

If $t \leqslant t_s$, the cumulative hazard function is equal to

$$\begin{aligned} H(t, x, z(t)) &= \int_0^t \lambda \, \exp(\beta z(u) + \eta' x) \, du \\ &= \int_0^t \lambda \, \exp(\beta u + \eta' x) \, du \\ &= \lambda \, \exp(\eta' x) \int_0^t \exp(\beta u) \, du \\ &= \lambda \, \exp(\eta' x) \left[\frac{1}{\beta} \exp(\beta u) \right]_0^t \\ &= \frac{\lambda \, \exp(\eta' x)}{\beta} \left[\exp(\beta t) - 1 \right]. \end{aligned}$$

Consequently, the inverse cumulative hazard function is

$$H^{-1}(v) = \frac{1}{\beta} \log \left(1 + \frac{\beta v}{\lambda \, \exp(\eta' x)} \right).$$

Therefore, t actually follows the Gompertz distribution with a scale parameter of $\lambda \exp(\eta' x)$ and a shape parameter of β. Therefore, if $t \leqslant t_s$, the event time can be generated as

$$T = \frac{1}{\beta} \log \left(1 + \frac{\beta(-\log(u))}{\lambda \, \exp(\eta' x)} \right), \text{ if } -\log(u) < \frac{\lambda \, \exp(\eta' x)}{\beta} \left[\exp(\beta t_s) - 1 \right],$$

where u is the realization of a $U(0, 1)$ random variable.

A1: single-dose approach assuming Weibull distribution of baseline hazard

The log of the Weibull hazard function is linear in log(t) and the hazard function can be written as $h_0(t) = \lambda \gamma t^{\gamma - 1}$. Hence, the cumulative hazard function becomes

$$H(t|x, z(t)) = \int_0^t \lambda \gamma u^{\gamma - 1} \, \exp(\beta z(u) + \eta' x) \, du.$$

If $t \leqslant t_s$, the cumulative hazard function is equal to

$$\begin{aligned}
H(t,x,z(t)) &= \int_0^t \lambda\gamma u^{\gamma-1}\ \exp(\beta z(u) + \eta' x)\, du \\
&= \int_0^t \lambda\gamma u^{\gamma-1}\ \exp(\beta u + \eta' x)\, du \\
&= \lambda\gamma\ \exp(\eta' x) \int_0^t u^{\gamma-1}\ \exp(\beta u)\, du \\
&= \lambda\gamma \exp(\eta' x)\ \left[(-\beta)^{-\gamma}\Gamma\left(\gamma, -\beta u\right)\right]_0^t \\
&= \frac{\lambda\gamma\ \exp(\eta' x)}{(-\beta)^{\gamma}}\left[\Gamma\left(\gamma, -\beta t\right) - \Gamma\left(\gamma, 0\right)\right]
\end{aligned}$$

Consequently, the inverse cumulative hazard function is

$$H^{-1}(v) = -\frac{1}{\beta}\Gamma^{-1}\left[\gamma, \frac{(-\beta)^{\gamma} v}{\lambda\ \exp(\eta' x)} + \Gamma(\gamma, 0)\right]$$

where $\Gamma^{-1}(\gamma, f(x))$ represents the inverse upper incomplete gamma function. Therefore, an event time can be generated as

$$T = -\frac{1}{\beta}\Gamma^{-1}\left[\gamma, \frac{(-\beta)^{\gamma}(-\log(u))}{\lambda\ \exp(\eta' x)} + \Gamma(\gamma, 0)\right], \text{ if}$$
$$-\log(u) < \frac{\lambda\gamma\ \exp(\eta' x)}{(-\beta)^{\gamma}}\left[\Gamma\left(\gamma, -\beta t_s\right) - \Gamma\left(\gamma, 0\right)\right] \quad (0.1)$$

where u is the realization of a $U(0,1)$ random variable.

If $t > t_s$, the cumulative hazard function is equal to

$$\begin{aligned}
H(t,x,z(t)) &= \int_0^{t_s} \lambda\gamma u^{\gamma-1}\ \exp(\beta u + \eta' x) du + \int_{t_s}^t \lambda\gamma t_s^{\gamma-1}\ \exp(\beta t_s + \eta' x) du \\
&= \lambda\gamma\ \exp(\eta' x)\left(\frac{1}{(-\beta)^{\gamma}}\left(\Gamma\left(\gamma, -\beta t_s\right) - \Gamma\left(\gamma, 0\right)\right) + (t - t_s) t_s^{\gamma-1}\ \exp(\beta t_s)\right)
\end{aligned}$$

Consequently, the inverse cumulative hazard function is

$$H^{-1}(v) = \frac{v}{t_s^{\gamma-1}\lambda\gamma\ \exp(\beta t_s + \eta' x)} - \frac{\Gamma\left(\gamma, -\beta t_s\right) - \Gamma\left(\gamma, 0\right)}{t_s^{\gamma-1}(-\beta)^{\gamma}\ \exp(\beta t_s)} + t_s.$$

Therefore, an event time can be generated as

$$T = \frac{-\log(u)}{t_s^{\gamma-1}\lambda\gamma\, \exp(\beta t_s + \eta' x)} - \frac{\Gamma(\gamma, -\beta t_s) - \Gamma(\gamma, 0)}{t_s^{\gamma-1}(-\beta)^{\gamma}\, \exp(\beta t_s)} + t_s, \text{ if}$$

$$-\log(u) \geqslant \frac{\lambda\gamma\, \exp(\eta' x)}{(-\beta)^{\gamma}} \left[\Gamma(\gamma, -\beta t_s) - \Gamma(\gamma, 0)\right] \quad (0.2)$$

where u is the realization of a $U(0,1)$ random variable.

A2: single-dose approach assuming Gompertz distribution of baseline hazard

The log of the Gompertz hazard function is linear in t and the hazard function can be written as $h_0(t) = \lambda\, \exp(\alpha t)$. Hence, the cumulative hazard function becomes

$$H(t|x, z(t)) = \int_0^t \lambda\, \exp(\alpha u)\, \exp(\beta z(u) + \eta' x)\, du.$$

If $t \leqslant t_s$, the cumulative hazard function is equal to

$$\begin{aligned} H(t, x, z(t)) &= \int_0^t \lambda\, \exp(\alpha u)\, \exp(\beta z(u) + \eta' x)\, du \\ &= \int_0^t \lambda\, \exp(\alpha u)\, \exp(\beta u + \eta' x)\, du \\ &= \lambda\, \exp(\eta' x) \int_0^t \exp((\beta + \alpha) u)\, du \\ &= \lambda\, \exp(\eta' x) \left[\frac{1}{\beta + \alpha}\, \exp((\beta + \alpha) u)\right]_0^t \\ &= \frac{\lambda\, \exp(\eta' x)}{\beta + \alpha} \left[\, \exp((\beta + \alpha) t) - 1\right] \end{aligned}$$

Consequently, the inverse cumulative hazard function is

$$H^{-1}(v) = \frac{1}{\beta + \alpha} \log\left(1 + \frac{(\beta + \alpha) v}{\lambda\, \exp(\eta' x)}\right).$$

Therefore, an event time can be generated as

$$T = \frac{1}{\beta + \alpha} \log\left(1 + \frac{(\beta + \alpha)(-\log(u))}{\lambda\, \exp(\eta' x)}\right), \text{ if}$$

$$-\log(u) < \frac{\lambda\, \exp(\eta' x)}{\beta + \alpha} \left[\, \exp((\beta + \alpha) t_s) - 1\right] \quad (0.3)$$

where u is the realization of a $U(0,1)$ random variable.

If $t > t_s$, the cumulative hazard function is equal to

$$\begin{aligned} H(t,x,z(t)) &= \int_0^{t_s} \lambda\ ((\beta+\alpha)u + \eta' x)\, du + \int_{t_s}^{t} \lambda\ \exp((\beta+\alpha)t_s + \eta' x), du \\ &= \lambda\ \exp(\eta' x) \left(\frac{1}{\beta+\alpha} (\exp((\beta+\alpha)t_s) - 1) + (t - t_s)\ \exp((\beta+\alpha)t_s) \right) \end{aligned}$$

Consequently, the inverse cumulative hazard function is

$$H^{-1}(v) = \frac{v}{\lambda\ \exp((\beta+\alpha)t_s + \eta' x)} + \frac{1 -\ \exp((\beta+\alpha)t_s)}{(\beta+\alpha)\ \exp((\beta+\alpha)t_s)} + t_s.$$

Therefore, an event time can be generated as

$$\begin{aligned} T = \frac{-\log(u)}{\lambda\ \exp((\beta+\alpha)t_s + \eta' x)} + \frac{1 -\ \exp((\beta+\alpha)t_s)}{(\beta+\alpha)\ \exp((\beta+\alpha)t_s)} + t_s, \text{ if} \\ -\log(u) \geqslant \frac{\lambda\ \exp(\eta' x)}{\beta+\alpha}\, [\ \exp((\beta+\alpha)t_s) - 1] \end{aligned} \quad (0.4)$$

where u is the realization of a $U(0,1)$ random variable.

A3: multiple-dose approach assuming imperfect infusion adherence

In a multiple-dose setting, perfect adherence to the 8-weekly infusion schedule is not always assured. If the "zero-protection" threshold t_s is smaller than a dosing interval, then modifications of the derivations covered in Section 2.2.2 are needed when the next infusion occurs after t_s has passed.

As stated in Section 2.2.1, in a single-dose setting, for $t > t_s$, the cumulative hazard function is equal to

$$H(t,x,z(t)) = \frac{\lambda \exp(\eta' x)}{\beta} [\exp(\beta t) - 1] + \lambda \exp(\eta' x)(t - t_s) \exp(\beta t_s).$$

And, an event time can be generated as

$$T = \frac{-\log(u)}{\lambda \exp(\beta t_s + \eta' x)} + \frac{1 - \exp(\beta t_s)}{\beta \exp(\beta t_s)} + t_s, \text{ if}$$
$$-\log(u) \geqslant \frac{\lambda \exp(\eta' x)}{\beta}[\exp(\beta t_s) - 1], \quad (0.5)$$

where u is the realization of a $U(0,1)$ random variable.

As stated in Section 2.2.2, in a multiple-dose setting, for $t_k \leqslant t < t_{k+1}, k = 1...., m-1$, the cumulative hazard function is equal to

$$H(t, x, z(t)) = \frac{\lambda}{\beta} \exp(\eta' x) \left[\sum_{i=2}^{k} \exp(\beta(t_i - t_{i-1})) + \exp(\beta t - \beta t_k) - k \right].$$

And, the event time can be generated as

$$T = \frac{1}{\beta} \log \left(\exp(\beta t_k) \left(\frac{\beta(-\log(u))}{\lambda \exp(\eta' x)} - \sum_{i=2}^{k} \exp(\beta(t_i - t_{i-1})) + k \right) \right), \text{ if}$$
$$a \leqslant -\log(u) < b, \quad (0.6)$$

where,

$$a = \frac{\lambda}{\beta} \exp(\eta' x) \left(\sum_{i=2}^{k} \exp(\beta(t_i - t_{i-1})) - (k-1) \right),$$
$$b = \frac{\lambda}{\beta} \exp(\eta' x) \left(\sum_{i=2}^{k} \exp(\beta(t_i - t_{i-1})) + \exp(\beta t_{k+1} - \beta t_k) - k \right),$$

and, u is the realization of a $U(0,1)$ random variable.

Now consider $t_k + t_s \leqslant t < t_{k+1}$ in a multiple-dose setting, where all infusions up till the kth perfectly adhere to the 8-weekly schedule. The cumulative hazard function is equal to

$$\begin{aligned} H(t, x, z(t)) &= \int_0^t \exp(\beta z(u) + \eta' x) du \\ &= \lambda \exp(\eta' x) \int_0^t \exp(\beta z(u)) du \\ &= \lambda \exp(\eta' x) \left[\int_0^{t_k} \exp(\beta z(u)) du + \int_{t_k}^{t_k + t_s} \exp(\beta(u - t_k) du + \int_{t_k + t_s}^{t} \exp(\beta(t_s)) du \right] \\ &= \frac{\lambda}{\beta} \exp(\eta' x) \left[\sum_{i=2}^{k} \exp(\beta(t_i - t_{i-1})) + \exp(\beta(t_s)) + \beta(t - t_s - t_k) \exp(\beta(t_s)) - k \right]. \end{aligned}$$

And, the inverse cumulative function is

$$H^{-1}(u) = \frac{1}{\beta \exp(\beta t_s)} \left(\frac{\beta u}{\lambda \exp(\eta' x)} - \sum_{i=2}^{k} \exp(\beta(t_i - t_{i-1})) - \exp(\beta t_s) + k \right) + t_s + t_k.$$

Therefore, an event time can be generated as

$$T = \frac{1}{\beta \exp(\beta t_s)} \left(\frac{\beta(-\log(u))}{\lambda \exp(\eta' x)} - \sum_{i=2}^{k} \exp(\beta(t_i - t_{i-1})) - \exp(\beta t_s) + k \right) + t_s + t_k, \text{ if}$$
$$a \leqslant -\log(u) < b, \quad (0.7)$$

where,

$$a = \frac{\lambda}{\beta} \exp(\eta' x) \left[\sum_{i=2}^{k} \exp(\beta(t_i - t_{i-1})) + \exp(\beta t_s) - k \right],$$
$$b = \frac{\lambda}{\beta} \exp(\eta' x) \left[\sum_{i=2}^{k} \exp(\beta(t_i - t_{i-1})) + \exp(\beta t_{k+1} - \beta t_k) - k \right],$$

and, u is the realization of a $U(0,1)$ random variable.

If more infusions continue to be given after a violation of the infusion schedule, and $t_{k+n} \leqslant t < t_{k+n} + t_s$ and $t_k \leqslant t_k + t_s < t_{k+1}$, then the cumulative hazard function is equal to

$$\begin{aligned}
H(t, x, z(t)) =& \int_0^t \exp(\beta z(u) + \eta' x) du \\
=& \lambda \exp(\eta' x) \int_0^t \exp(\beta z(u)) du \\
=& \lambda \exp(\eta' x) \left(\int_0^{t_k} \exp(\beta z(u) du + \int_{t_k}^{t_k + t_s} \exp(\beta(u - t_k) du \right. \\
& \left. + \int_{t_k + t_s}^{t_{k+1}} \exp(\beta(t_s)) du + \int_{t_{k+1}}^{t} \exp(\beta z(u)) du \right) \\
=& \frac{\lambda}{\beta} \exp(\eta' x) \left[\sum_{i=2}^{k} \exp(\beta(t_i - t_{i-1})) + \sum_{i=k+2}^{k+n} \exp(\beta(t_i - t_{i-1})) \right. \\
& \left. + \exp(\beta t - \beta t_{k+n}) + \beta(t_{k+1} - t_s - t_k) \exp(\beta t_s) + \exp(\beta t_s) - (k+n) \right].
\end{aligned}$$

And, the inverse cumulative function is

$$H^{-1}(u) = \frac{1}{\beta}\log\left(\exp(\beta t_{k+n})\left(\frac{\beta(u)}{\lambda\exp(\eta' x)} - \sum_{i=2}^{k}\exp(\beta(t_i - t_{i-1})) - \sum_{i=k+2}^{k+n}\exp(\beta(t_i - t_{i-1}))\right.\right.$$
$$\left.\left. - \beta(t_{k+1} - t_s - t_k)\exp(\beta t_s) - \exp(\beta t_s) + (k+n)\right)\right).$$

Therefore, an event time can be generated as

$$T = \frac{1}{\beta}\log\left(\exp(\beta t_{k+n})\left(\frac{\beta(-\log(u))}{\lambda\exp(\eta' x)} - \sum_{i=2}^{k}\exp(\beta(t_i - t_{i-1})) - \sum_{i=k+2}^{k+n}\exp(\beta(t_i - t_{i-1}))\right.\right.$$
$$\left.\left. - \beta(t_{k+1} - t_s - t_k)\exp(\beta t_s) - \exp(\beta t_s) + (k+n)\right)\right), \text{ if } a \leqslant -\log(u) < b, \text{ where}$$
$$a = \frac{\lambda}{\beta}\exp(\eta' x)\left[\sum_{i=2}^{k}\exp(\beta(t_i - t_{i-1})) + \sum_{i=k+2}^{k+n}\exp(\beta(t_i - t_{i-1}))\right.$$
$$\left. + \beta(t_{k+1} - t_s - t_k)\exp(\beta t_s) + \exp(\beta t_s) - (k+n-1)\right],$$
$$b = \frac{\lambda}{\beta}\exp(\eta' x)\left[\sum_{i=2}^{k}\exp(\beta(t_i - t_{i-1})) + \sum_{i=k+2}^{k+n}\exp(\beta(t_i - t_{i-1}))\right.$$
$$\left. + \exp(\beta t_{k+n+1} - \beta t_{k+n}) + \beta(t_{k+1} - t_s - t_k)\exp(\beta t_s) + \exp(\beta t_s) - (k+n)\right],$$

and u is the realization of a $U(0,1)$ random variable.

The strategies described above can be extrapolated to settings where multiple violations to the regular infusion schedule occur.

A4: illustration of the single-dose and multiple-dose approaches with alternative sample sizes, complementing Figures 2 and 3 in the main text.

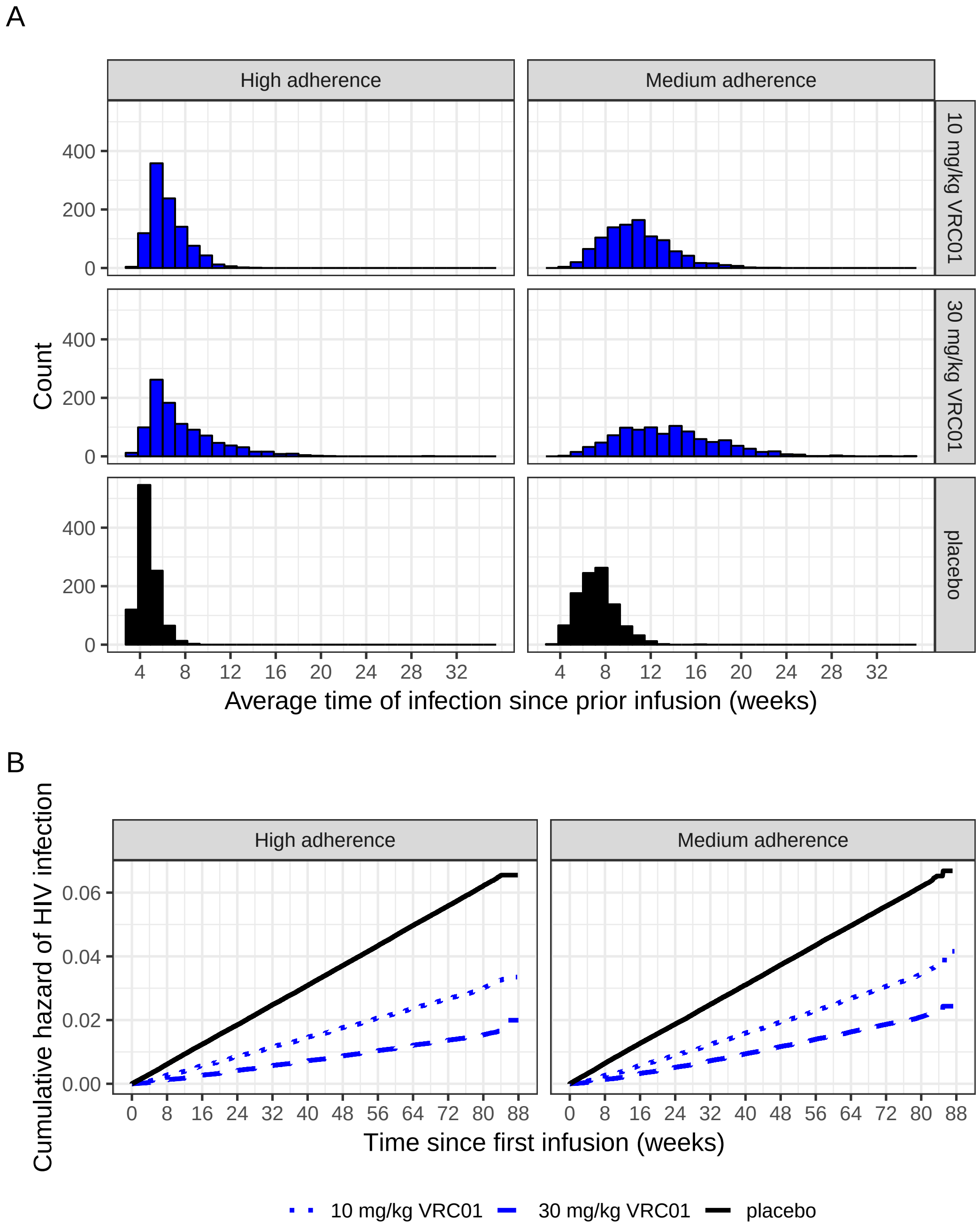

Figure 2-supplement: Distributions of simulated event times since prior infusion (Panel A) and cumulative hazard of HIV infection since the first infusion (Panel B)

under imperfect study adherences in AMP-like trials. The single-dose approach is used in these simulations of 1000 trials, each with a total of $n = 2100$ participants randomized to receive ten 8-weekly infusions of 10 mg/Kg VRC01, 30 mg/Kg VRC01 or placebo in a 1:1:1 ratio. The high and medium adherence scenarios assume 2% and 10% of infusion visits missed, respectively. Additional assumptions are as follows: annual HIV incidence rate = 4% in the placebo group, $\beta = 0.03$ or HR= 2.32 per-28 days for both VRC01 dose groups, and zero-protection concentration threshold $s = 5$ mcg/mL.

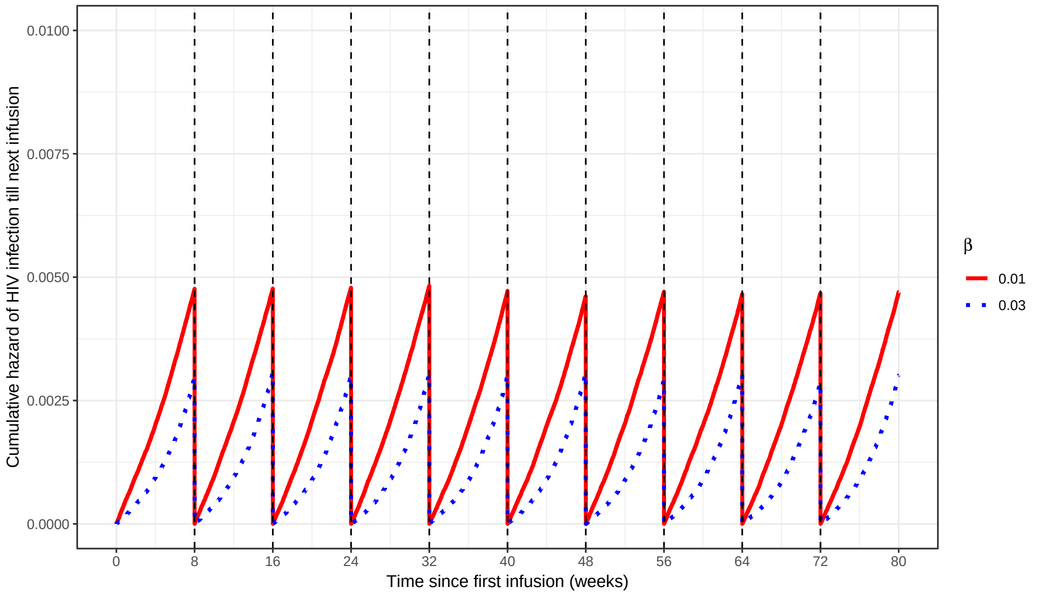

**Infusion:**	1	2	3	4	5	6	7	8	9	10	
**No. at risk:**											
$\beta_t = 0.01$	1400	1393	1387	1380	1373	1367	1361	1354	1348	1342	1335
$\beta_t = 0.03$	1400	1396	1392	1387	1383	1379	1375	1371	1367	1363	1359
**Cumulative No. of infections within each infusion interval:**											
$\beta_t = 0.01$	0	7	7	7	7	6	6	6	6	6	6
$\beta_t = 0.03$	0	4	4	4	4	4	4	4	4	4	4

Figure 3-supplement: Cumulative hazard of HIV infection within each infusion interval following ten 8-weekly IV infusions of VRC01 under perfect study adherence in a simulated trial of 1400 VRC01 recipients. Red lines are for $\beta = 0.01$ or $HR = 1.32$ per-28 days; blue lines are for $\beta = 0.03$ or HR$= 2.32$ per-28 days.

References

Austin, P.C. (2012). Generating survival times to simulate Cox proportional hazards models with time-varying covariates. *Statistics in Medicine.* **31(39)**, 4658.

Huang, Y., Zhang, L., Ledgerwood, J., Grunenberg, N., Bailer, R., Isaacs, A., Seaton, K., Mayer, K.H., Capparelli, E., Corey, L., Gilbert, P.B. (2017). Population Pharmacokinetics Analysis of VRC01, a Broadly Neutralizing HIV-1 Monoclonal Antibody, in Healthy Adults. *mAbs.* **9(5)**, 792-800.
